# Systemic neutrophil activation and *N*‐formyl methionine‐formyl peptide receptor‐1 signaling define inflammatory endotypes in rheumatoid arthritis‐associated lung involvement

**DOI:** 10.1111/joim.70030

**Published:** 2025-10-14

**Authors:** Jia Shi, Chen Yu, Dan Ke, Xueting Yuan, Yiyun Pang, Yang Wu, Ting Wang, Ryan Stultz, Xiaomin Liu, Xinping Tian, Mengtao Li, Qian Wang, M. Kristen Demoruelle, Joshua J. Solomon, Christian Lood

**Affiliations:** ^1^ Division of Rheumatology University of Washington Seattle Washington USA; ^2^ Department of Rheumatology and Clinical Immunology Peking Union Medical College Hospital Chinese Academy of Medical Sciences & Peking Union Medical College Beijing China; ^3^ National Clinical Research Center for Dermatologic and Immunologic Diseases (NCRC‐DID) Ministry of Science & Technology Beijing China; ^4^ State Key Laboratory of Complex Severe and Rare Diseases Key Laboratory of Rheumatology and Clinical Immunology Ministry of Education Beijing China; ^5^ Department of Rheumatology Shunyi District Hospital Beijing China; ^6^ State Key Laboratory of Common Mechanism Research for Major Diseases Key Laboratory of Rheumatology and Clinical Immunology Ministry of Education Beijing China; ^7^ Division of Rheumatology University of Colorado Denver Denver Colorado USA; ^8^ National Jewish Health Center for Interstitial Lung Disease Denver Colorado USA

**Keywords:** FPR1, interstitial lung disease, mitochondria, neutrophil, rheumatoid arthritis

## Abstract

**Background:**

Neutrophil activation plays a crucial role in the pathogenesis of rheumatoid arthritis (RA), but its involvement in RA‐associated interstitial lung disease (RA‐ILD) remains unclear. This study investigated the involvement of *N*‐formyl methionine (fMET) and its receptor formyl peptide receptor‐1 (FPR1) in neutrophil‐mediated inflammation in RA‐ILD.

**Methods:**

Plasma and sputum levels of fMET and neutrophil activation markers were measured by ELISA in two cohorts (*n* = 269 and 314) spanning multiple disease subgroups. Neutrophil activation was assessed by flow cytometry following plasma stimulation, with or without FPR1 inhibitors.

**Results:**

Calprotectin levels were significantly elevated in both plasma and sputum of RA‐ILD patients compared to controls and RA‐noILD patients (*p *< 0.05 for all analyses) and were negatively correlated with pulmonary function in RA (forced vital capacity [FVC], *r* = −0.39, *p *= 0.0002; diffusing capacity for carbon monoxide [DLCO], *r* = −0.39, *p *= 0.001). Plasma fMET levels were higher in RA‐ILD patients compared to healthy controls (*p* < 0.0001) as well as compared to RA‐noILD patients (*p *< 0.01), with a significant inverse correlation to pulmonary function in RA patients (FVC, *r* = −0.42, *p *< 0.0001; DLCO, *r* = −0.31, *p *= 0.01). Plasma from RA‐ILD patients induced neutrophil activation through FPR1‐dependent mechanisms (*p *< 0.0001). Hierarchical clustering identified reproducible subgroups defined by fMET and calprotectin, with the high‐fMET cluster enriched for RA‐ILD and associated with lower DLCO (*p *< 0.05).

**Conclusions:**

The fMET–FPR1 axis is associated with neutrophil activation in RA‐ILD and defines inflammatory endotypes associated with lung impairment. Neutrophil‐based biomarkers may enable early risk stratification and provide rationale for targeting the fMET–FPR1 axis in RA‐ILD.

## Graphical abstract

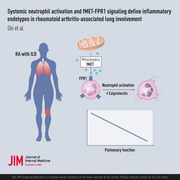

## Introduction

Rheumatoid arthritis (RA) is one of the most common systemic autoimmune diseases characterized by progressive joint destruction and extra‐articular manifestations, among which interstitial lung disease (ILD) is the most common, and carries high morbidity and mortality [1, 2, 3, 4]. However, the mechanisms of ILD in RA are poorly understood, and reliable biomarkers to identify high‐risk patients or inflammatory endotypes in RA remain lacking.

Neutrophils are central to RA pathogenesis, contributing not only to joint inflammation but also to systemic manifestations, including lung involvement. Activated neutrophils release calprotectin and form neutrophil extracellular traps (NETs), which amplify autoimmunity and tissue injury. In RA, neutrophils attack joint structures, causing pain, inflammation, and loss of function [5]. Recent studies have shown elevated levels of NETs and neutrophil activation markers such as calprotectin in the plasma of RA patients [6]. Interestingly, these markers, when measured at disease onset, correlate with significant disease progression, including joint erosion, space narrowing, and extra‐articular manifestations. This highlights neutrophil activation as an early event in RA pathogenesis that is associated with poor prognosis. Moreover, neutrophil‐derived molecules, including neutrophil elastase (NE) and histones, can perpetuate autoantigen exposure, trigger adaptive immune responses, and promote autoantibody production, leading to systemic inflammation that affects multiple organs, including the lungs [7]. NETs from patients with RA‐ILD have been shown to enhance the fibrotic potential of pulmonary fibroblasts in vitro, linking neutrophil activity directly to lung fibrosis [8]. Furthermore, neutrophil‐derived biomarkers and anti‐citrullinated protein antibodies (ACPAs) have been detected in the sputum of RA patients, further substantiating the connection between neutrophil activation and lung involvement [9]. However, the mechanisms driving neutrophil activation in the lung remain unclear.

One significant neutrophil agonist is *N*‐formyl methionine (fMET) peptides, derived from bacteria or mitochondria. Acting through the formyl peptide receptor‐1 (FPR1), fMET is a potent chemoattractant that activates a range of neutrophil functions, leading to inflammation and tissue damage [10, 11, 12]. We recently observed elevated levels of fMET in RA patients, which correlated with disease activity [13]. However, the role of FPR1 signaling in RA‐ILD remains unexplored.

Previous literature has highlighted the beneficial effects of targeting FPR1 in inflammatory and fibrotic conditions. FPR1 deficiency has been shown to reduce ischemia‐reperfusion damage to the heart [14], acute endotoxin–induced lung injury [15], and smoking‐induced lung emphysema [16]. Importantly, FPR1 appears to be uniquely implicated in lung fibrosis [17]. FPR1‐deficient mice are protected from bleomycin‐induced pulmonary fibrosis, with impaired neutrophil recruitment to the lungs. In contrast, neutrophil recruitment to the liver and kidney remains unaffected, and fibrosis in these organs develops normally, underscoring the tissue‐specific role of FPR1 in driving pulmonary fibrosis.

Given these findings, we hypothesize that fMET and downstream neutrophil activation may play a role in RA‐ILD pathogenesis. In this study, we investigated the clinical utility of fMET and neutrophil activation markers in RA‐ILD patients and examined the role of fMET–FPR1 signaling in neutrophil activation, aiming to provide new mechanistic and translational insights.

## Materials and methods

### Patient cohorts and ethical statements

Plasma samples from two independent RA cohorts were analyzed in the current study. The demographic data and clinical characteristics are detailed in Table 1, and the available biomarker and clinical variables in each cohort are summarized in Table S1.

**Table 1:** Demographic and clinical data of subject.

	Cohort I (*n* = 269)						Cohort II (*n* = 314)		
Variables	RA‐ILD (*n* = 50)	RA‐noILD (*n* = 49)	COPD (*n* = 50)	IPF (*n* = 50)	SSc (*n* = 20)	HC (*n* = 50)	RA‐ILD (*n* = 121)	RA‐noILD (*n* = 98)	HC (*n* = 95)
Age, mean (SD)	67.9 (9.2)	54.2 (10.2)	65.5 (8.9)	68.9 (6.9)	60.6 (9.6)	60.4 (10.2)	63.4 (10.5)	49.7 (12.7)	50.4 (8.9)
Female, *n* (%)	29 (58)	41 (83.7)	37 (74)	12 (24)	15 (75)	32 (64)	72 (59.5)	83 (84.7)	68 (71.6)
**Serology**									
Positive RF, *n* (%)	15 (39.5%)	21 (55.3%)	/	/	/	/	107 (93.0%)	80 (96.4%)	/
RF (IU/mL) median (IQR)	/	/	/	/	/	/	153.8 (65.5, 429.5)	107.6 (54.1, 246.9)	/
Positive ACPA, *n* (%)	11 (52.4%)	12 (50.0%)	/	/	/	/	105 (92.1%)	73 (88.0%)	/
ACPA (U/mL) median (IQR)	/	/	/	/	/	/	201.0 (109.9, 504.3)	200.0 (115.1, 1394.0)	/
Seropositive, *n* (%)	20 (74.1%)	23 (76.7%)	/	/	/	/	114 (99.1%)	83 (100%)	/
Disease activity									
DAS28‐CRP, median (IQR)	/	/	/	/	/	/	3.01 (1.78, 4.25)	2.65 (1.69, 5.45)	/
PGA, median (IQR)	/	/	/	/	/	/	30 (11, 60)	20 (5, 60)	/
**Race, *n* (%)**									
White	28 (56%)	28 (57%)	35 (70%)	15 (30%)	12 (60%)	42 (84%)	0	0	0
Asian	2 (4%)	2 (4%)	1 (2%)	0	0	1 (2%)	121 (100%)	98 (100%)	95 (100%)
Black	0	3 (6%)	2 (4%)	0	0	2 (4%)	0	0	0
Unknown	20 (40%)	16 (33%)	12 (24%)	35 (70%)	8 (40%)	5 (10%)	0	0	0
**Baseline pulmonary function**									
% FVC predicted, median (IQR)	71 (63, 88)	90 (83, 103)	70 (58, 82)	66 (50, 76)	81 (58, 93)	/	92 (80, 105)	/	/
% DLCO predicted, median (IQR)	63 (50, 77)	92 (86, 106)	71 (55, 82)	42 (32, 55)	63 (47, 80)	/	66 (52, 82)	/	/

*Note*: Percentages were calculated based on the number of patients with available data.

Abbreviations: ACPA, anti‐citrullinated protein antibody; COPD, chronic obstructive pulmonary disease; DLCO, diffusing capacity for carbon monoxide; FVC, forced vital capacity; IPF, idiopathic pulmonary fibrosis; RA–ILD, rheumatoid arthritis–interstitial lung disease; SSc, systemic sclerosis–related.

Cohort I consisted of participants from the University of Colorado and National Jewish Health, including plasma samples from the following groups: (1) RA‐ILD (*n* = 50), (2) RA‐noILD (*n* = 49), (3) chronic obstructive pulmonary disease (COPD, *n* = 50), (4) idiopathic pulmonary fibrosis (IPF, *n* = 50), (5) systemic sclerosis–related ILD (SSc‐ILD, *n* = 20), and (6) healthy controls (*n* = 50). Pulmonary function tests included forced vital capacity (FVC) and diffusing capacity for carbon monoxide (DLCO). The percentages of predicted values were used for all correlation analyses in our study. DLCO progression (%/year) was calculated as [(DLCO_follow‐up_ − DLCO_baseline_)/years of follow‐up] × 100, and FVC progression (%/year) was calculated using the same formula. For the RA patients, sputum samples were also collected. Cohort II was sourced from Peking Union Medical College Hospital, China, comprising plasma samples from (1) RA‐ILD (*n* = 121), (2) RA‐noILD (*n* = 98), and (3) healthy controls (*n* = 95). The study was approved by the ethics boards of University of Washington (#3100) and PUMCH (#I‐23PJ895), and informed consent was obtained from all participants in accordance with the Helsinki Declaration.

### ELISA‐based methods

Levels of calprotectin (S100A8/A9) were analyzed using a commercial ELISA kit according to manufacturer's instructions (R&D Systems). Plasma levels of human fMET were analyzed using a commercial ELISA kit according to manufacturer's instructions (My BioSource Inc.).

Circulating NETs were quantified using an NE‐DNA ELISA, as described previously [18]. Briefly, high binding 96 well ELISA microplates were coated with anti‐NE antibody (4 mg/mL; Calbiochem) overnight at 4°C, followed by blocking with 1% bovine serum albumin (BSA) in phosphate‐buffered saline (PBS) for 2 h at RT. After blocking, plasma samples and sputum samples (1:10 and 1:200 dilution in 1% BSA in PBS with 2 mM EDTA for plasma and sputum, respectively) were added and incubated overnight at 4°C. Anti‐DNA‐HRP from Cell Death Detection ELISA kit (clone MCA‐33; Roche) was added as a secondary antibody for 2 h at RT. The reaction was developed with 3,3′,5,5′ tetramethylbenzidine (BD Biosciences) and ended by the addition of 2N sulfuric acid. All ELISA assays were measured at 450 nm (Synergy 2, BioTek).

### Neutrophil activation assay

Neutrophils were isolated from healthy subjects by layering heparinized blood on Polymorphprep (Axis‐Shield) density gradient, according to the manufacturer's instructions. Red blood cells were lysed with RBC lysis buffer (BioLegend). For in vitro assays, neutrophils were resuspended in serum‐free RPMI‐1640 medium (ThermoFisher). Neutrophils were plated at 2 × 10^6^ cells/mL and were incubated with or without selective inhibitors of FPR1, anti‐FPR1 (150 µg/mL), or cyclosporine H (CsH, 5 µM) for 30 min before the addition of stimuli. As stimuli, plasma and sputum from Cohort I were used and incubated with the neutrophils for an additional 3 h. Neutrophils without pre‐treatment and stimuli were used as negative controls. Neutrophil activation was assessed by analyzing cell surface expression of CD66b (clone G10F5, BioLegend) and CD11b (clone CBRM1/5, BioLegend) by flow cytometry. Data were analyzed by FlowJo (Tree Star Inc.), and results were presented as relative mean fluorescent intensity (MFI) % of CD66b and CD11b relative to healthy controls (set as 100%). % Inhibition was calculated as [1 − (plasma‐induced activation marker MFI in presence of anti‐FPR1 − negative control activation marker MFI)/(plasma‐induced activation marker MFI − negative control activation marker MFI)] × 100.

### Cluster analysis

Hierarchical cluster analysis was performed using Ward's method based on Euclidean distances. Plasma levels of calprotectin and fMET were used as the two variables for clustering the RA patients. The optimal number of clusters was determined using the silhouette method.

### Statistical methods

For sample sets with a non‐Gaussian distribution, the Mann–Whitney *U* test, Wilcoxon's paired test, and Spearman's correlation test were used. For comparisons involving six groups, the Kruskal–Wallis test was performed, followed by Dunn's multiple comparisons test with Bonferroni correction for post hoc analysis. To account for potential confounding by serology or disease activity, adjusted linear regression models and partial correlation analyses were performed. GraphPad Prism, SPSS software, and R software (version 4.3.3) were used for the analysis. *p* values <0.05 were considered significant.

## Results

### Elevated concentrations of neutrophil activation markers in plasma and sputum of RA‐ILD

**Fig. 1:**
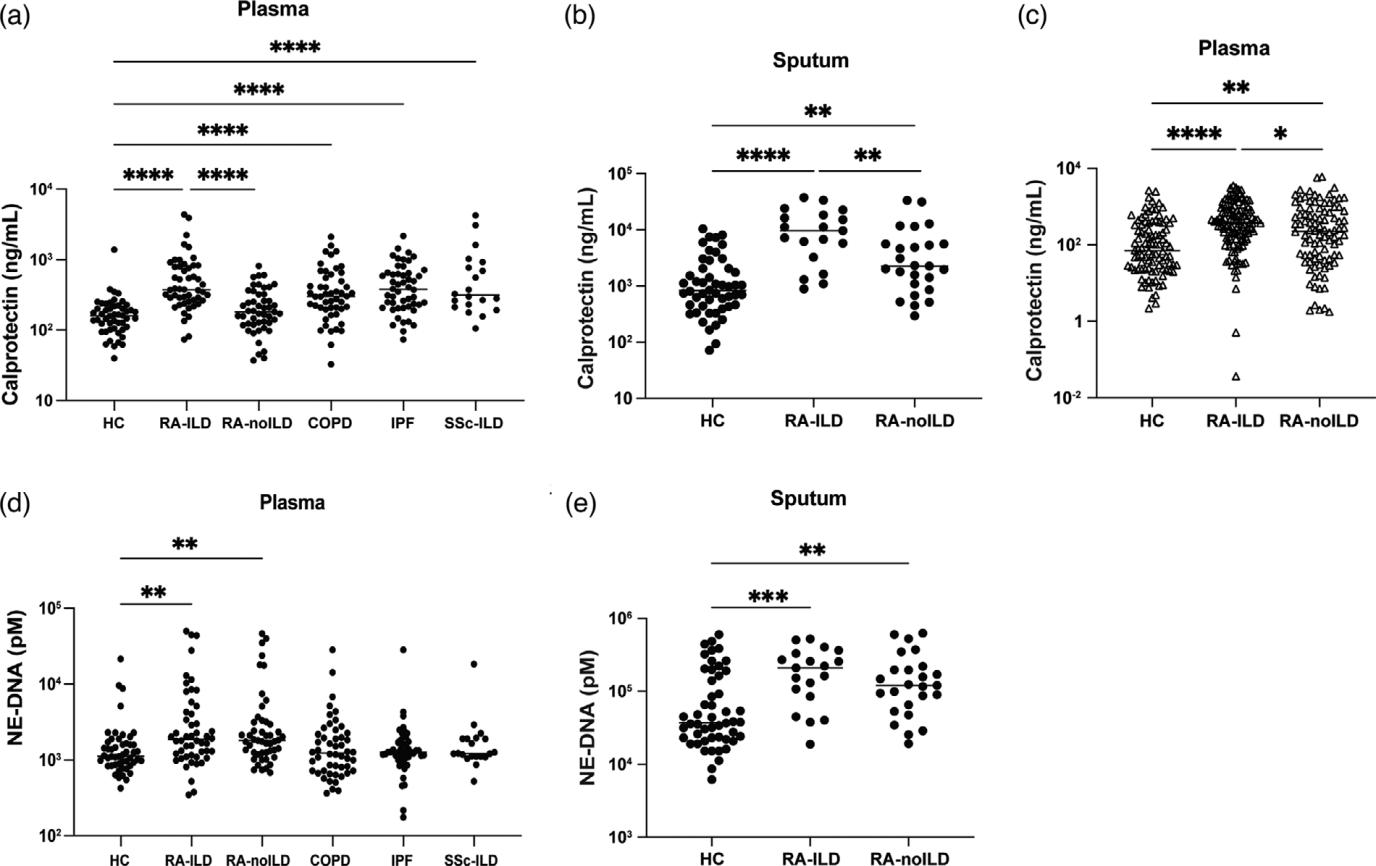
Levels of neutrophil activation markers in plasma and sputum of rheumatoid arthritis–interstitial lung disease (RA‐ILD) patients: (a and b) levels of calprotectin (S100A8/A9) and (d and e) neutrophil extracellular traps (NETs, measured as neutrophil elastase–DNA complexes, NE‐DNA) were analyzed by ELISA in plasma and sputum from Cohort I; (c) levels of calprotectin in plasma in Cohort II. Each symbol represents a single subject. Y‐axis is plotted on a log_10_ scale. Statistical analyses by the Kruskal–Wallis test, and the Mann–Whitney U test with *p < 0.05, **p < 0.01, ***p < 0.001, and ****p < 0.0001. COPD, chronic obstructive pulmonary disease; IPF, idiopathic pulmonary fibrosis; SSc–ILD, systemic sclerosis–related interstitial lung disease.

To investigate neutrophil activation in RA‐ILD, we analyzed calprotectin (S100A8/A9) levels in plasma samples from two independent cohorts, comparing them with levels in disease controls and healthy controls. In Cohort I, plasma levels of calprotectin were significantly increased in RA‐ILD, COPD, IPF, and SSc‐ILD as compared to healthy controls (*p *< 0.0001 for all groups, Fig. 1a). Additionally, RA‐ILD showed significantly higher levels of calprotectin as compared to RA‐noILD patients (*p *< 0.0001). Similarly, sputum levels of calprotectin were markedly higher in RA‐ILD compared to healthy controls (*p *< 0.0001) and RA‐noILD (*p *= 0.0082) (Fig. 1b). In Cohort II, plasma levels of calprotectin were increased both in RA‐ILD and RA‐noILD groups compared to healthy controls (*p *< 0.0001 and *p *= 0.0035, respectively, Fig. 1c) with higher levels found in the RA‐ILD group compared to the RA‐noILD group (*p *= 0.0289).

Given prior links between calprotectin, disease activity, and serology in RA [6], we examined whether these factors accounted for the observed ILD‐calprotectin association. In Cohort I, where serology data were available, RA‐ILD patients continued to display significantly higher calprotectin after adjustment for serostatus (adjusted geometric mean ratio ≈2.1, 95% CI 1.40–3.09, *p* = 0.0005; Table S2). In Cohort II, where disease activity measures were available, RA‐ILD patients similarly exhibited higher calprotectin after adjustment for DAS28‐CRP and/or PGA (adjusted geometric mean ratios 2.1–2.8, all *p* < 0.02; Table S2). These results demonstrate that the elevation of calprotectin in RA‐ILD is not attributable to differences in autoantibody status or RA disease activity. It should be noted that disease activity data were largely unavailable in Cohort I, and nearly all patients in Cohort II were seropositive, precluding simultaneous adjustment for both variables.

In Cohort I, both plasma and sputum NE‐DNA levels were elevated in RA‐ILD (*p *= 0.0074 and *p *= 0.0010, respectively) and RA‐noILD (*p *= 0.0082 and *p *= 0.0039, respectively) compared to healthy controls (Fig. 1d,e).

However, sputum levels of calprotectin and NE‐DNA did not correlate with their plasma counterparts in RA‐ILD, RA‐noILD, or the total RA group (all *p* > 0.05; Table S3), suggesting that local airway inflammation may be partly independent of systemic neutrophil activation.

These findings demonstrate significant neutrophil activation in the plasma and sputum of RA patients, with RA‐ILD showing greater activation than RA‐noILD.

### Association of plasma neutrophil activation markers with pulmonary function and disease progression

**Fig. 2:**
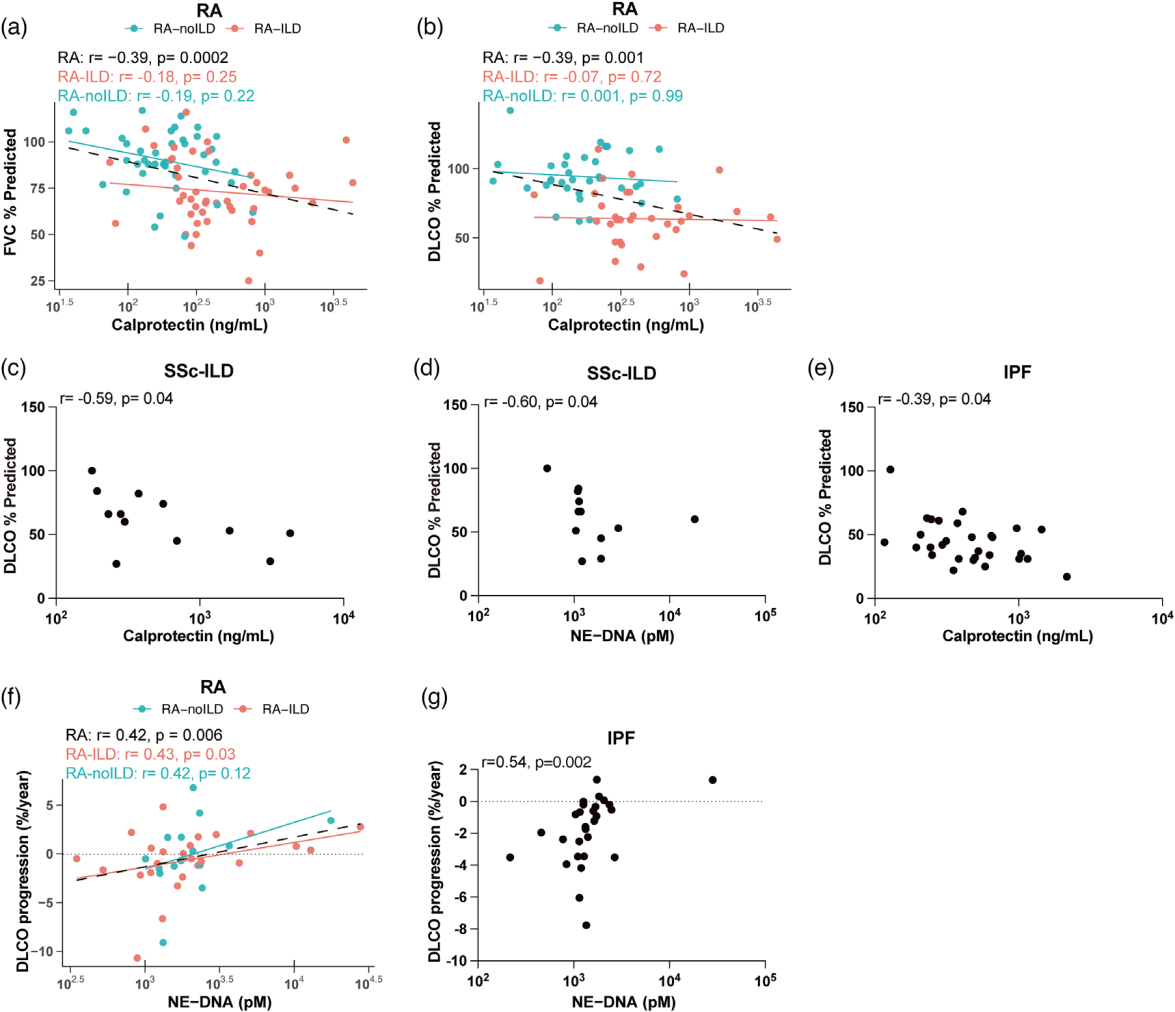
Association between neutrophil activation markers in plasma and pulmonary function: (a and b) correlation of calprotectin levels with % predicted forced vital capacity (FVC) (a) and % predicted diffusing capacity for carbon monoxide (DLCO) (b) in rheumatoid arthritis (RA); (c and d) correlation of calprotectin (c) and neutrophil elastase (NE)‐DNA (d) levels with % predicted DLCO in systemic sclerosis–related (SSc)–interstitial lung disease (ILD); (e) correlation of calprotectin levels with DLCO in idiopathic pulmonary fibrosis (IPF); (f and g) correlation of NE‐DNA levels with DLCO progression in RA (f) and IPF (g). X‐axes are shown on a log_10_ scale. Statistical analyses by Spearman's correlation.

We next examined the relationship between pulmonary function and neutrophil activation markers. In the total RA cohort from Cohort I, plasma calprotectin levels exhibited a significant negative correlation with pulmonary function, as assessed by FVC (*r* = −0.39, *p* = 0.0002) and DLCO (*r* = −0.39, *p* = 0.001) (Fig. 2a,b; Table S4). These associations remained significant after adjustment for serological status (Table S5). Similar correlations were observed in the SSc‐ILD and IPF groups. Plasma calprotectin levels showed a significant negative correlation with DLCO in both conditions (SSc‐ILD: *r* = −0.59, *p *= 0.044; IPF: *r* = −0.39, *p *= 0.036; Fig. 2c,e). Despite the limited sample size in the SSc‐ILD group, NE‐DNA levels also exhibited a significant negative correlation with DLCO (*r* = −0.60, *p* = 0.041) (Fig. 2d). In contrast, when restricting the analysis to RA‐ILD patients, no significant correlations with pulmonary function were observed in either Cohort I or Cohort II (Fig. 2a,b; Table S4). This lack of association persisted after adjustment for serology in Cohort I and for disease activity in Cohort II (Tables S5 and S6).

To investigate whether baseline neutrophil activation marker levels could predict pulmonary function progression, we analyzed follow‐up data. Interestingly, plasma levels of NE‐DNA at baseline correlated positively with DLCO progression in RA‐ILD, total RA cohort, and IPF groups (RA‐ILD: *r* = 0.43, *p *= 0.032; RA: *r* = 0.42, *p *= 0.006; IPF: *r* = 0.54, *p *= 0.002; Fig. 2f,g). This finding suggests that higher baseline NE‐DNA levels were associated with better pulmonary function at follow‐up. Importantly, this association with DLCO progression remained significant after adjustment for seropositivity (Table S7).

### Plasma fMET levels in lung disease groups and correlation with pulmonary function

**Fig. 3:**
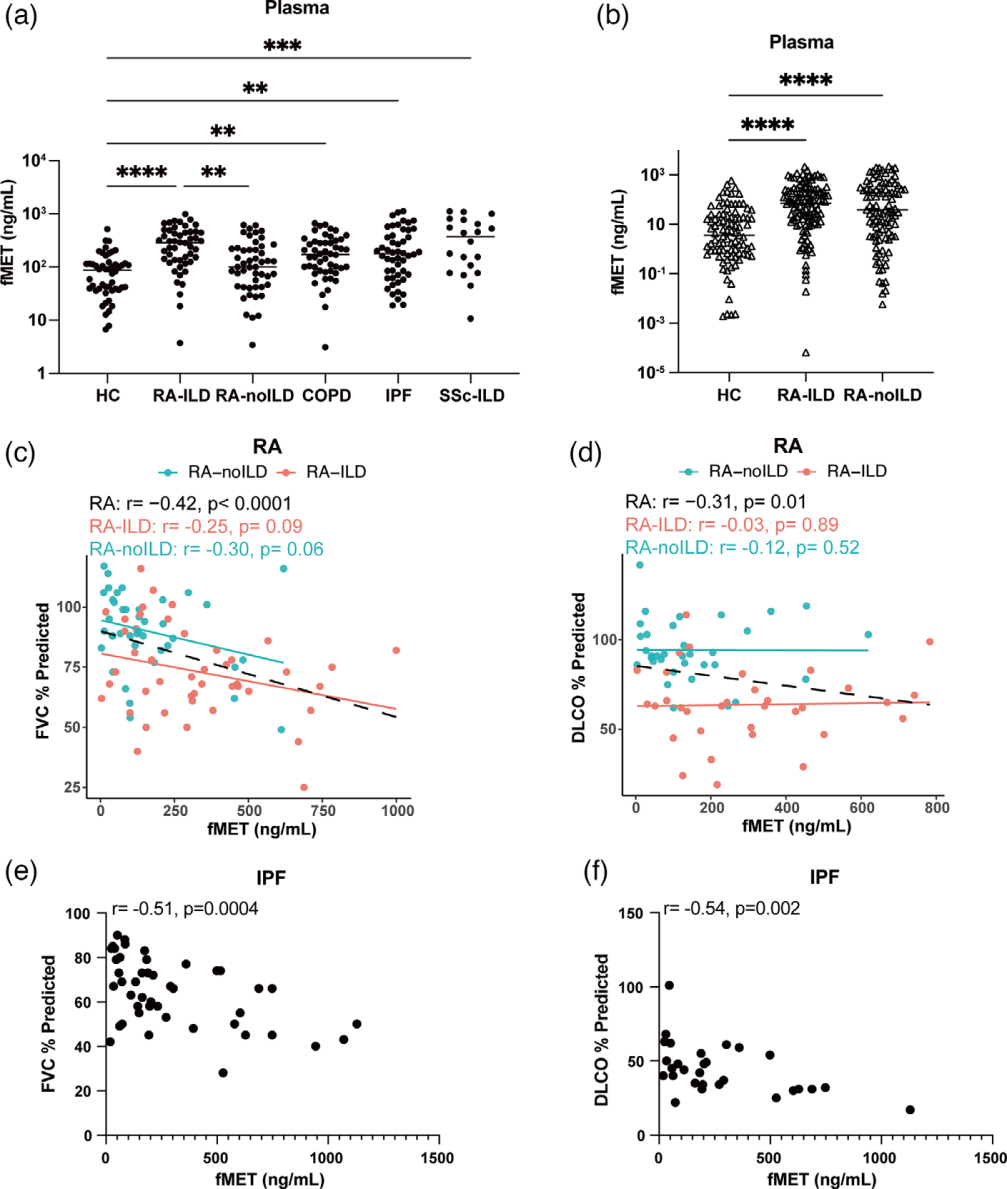
Plasma levels of N‐formyl methionine (fMET) in patients with lung disease: (a) plasma levels of fMET in healthy controls and patients with rheumatoid arthritis (RA), chronic obstructive pulmonary disease (COPD), idiopathic pulmonary fibrosis (IPF), and systemic sclerosis–related (SSc)–interstitial lung disease (ILD); (b) plasma levels of fMET in Cohort II; (c, d) correlation of fMET levels with % predicted forced vital capacity (FVC) (c) and % predicted diffusing capacity for carbon monoxide (DLCO) (d) in rheumatoid arthritis (RA); (e and f) correlation of fMET levels and % predicted with FVC (e), and % predicted DLCO (f) in IPF. Statistical analyses by the Kruskal–Wallis test, the Mann–Whitney U test, and Spearman's correlation with *p < 0.05, **p < 0.01, ***p < 0.001, and ****p < 0.0001.

As depicted in Fig. 3a, in Cohort I, levels of fMET were elevated in all lung disease groups (*p *< 0.01 for all groups) as compared to healthy individuals. Notably, the fMET levels in the RA‐ILD group were significantly higher than those in the RA‐noILD group (*p* = 0.0031). Similarly, in Cohort II, levels of fMET were elevated in both RA‐ILD and RA‐noILD as compared to healthy individuals (*p* < 0.0001), but there was no difference in fMET levels observed between the RA‐ILD and RA‐noILD groups in Cohort II (*p* = 0.631) (Fig. 3b). Because previous study has shown that fMET levels in RA may be influenced by serological status and disease activity [13], we performed adjusted analyses, which yielded consistent results after adjustment for serology in Cohort I and for disease activity in Cohort II (Table S8).

In Cohort I, plasma fMET levels demonstrated a significant negative correlation with FVC (*r* = −0.42, *p *< 0.001) and DLCO (*r* = −0.31, *p* = 0.010) (Fig. 3c,d). By contrast, within the RA‐ILD subgroup, no significant correlations were observed. When patients were categorized using the healthy control‐derived threshold (>mean + 2SD), RA patients with high fMET had significantly lower FVC compared with those with low fMET (*p* = 0.015; Fig. S1a). These associations remained significant after adjustment for serological status (Table S9).

In Cohort II, which included RA‐ILD patients with available pulmonary function tests, neither correlation analyses nor adjusted models yielded significant associations between fMET and lung function (Tables S4 and S10).

Similar results were seen in the IPF group, where plasma fMET levels correlated inversely with pulmonary function (FVC: *r* = −0.51, *p *= 0.0004; DLCO: *r* = −0.54, *p *= 0.002) (Fig. 3e,f).

### Plasma fMET induces neutrophil activation in an FPR1‐dependent manner in patients with RA‐ILD

**Fig. 4:**
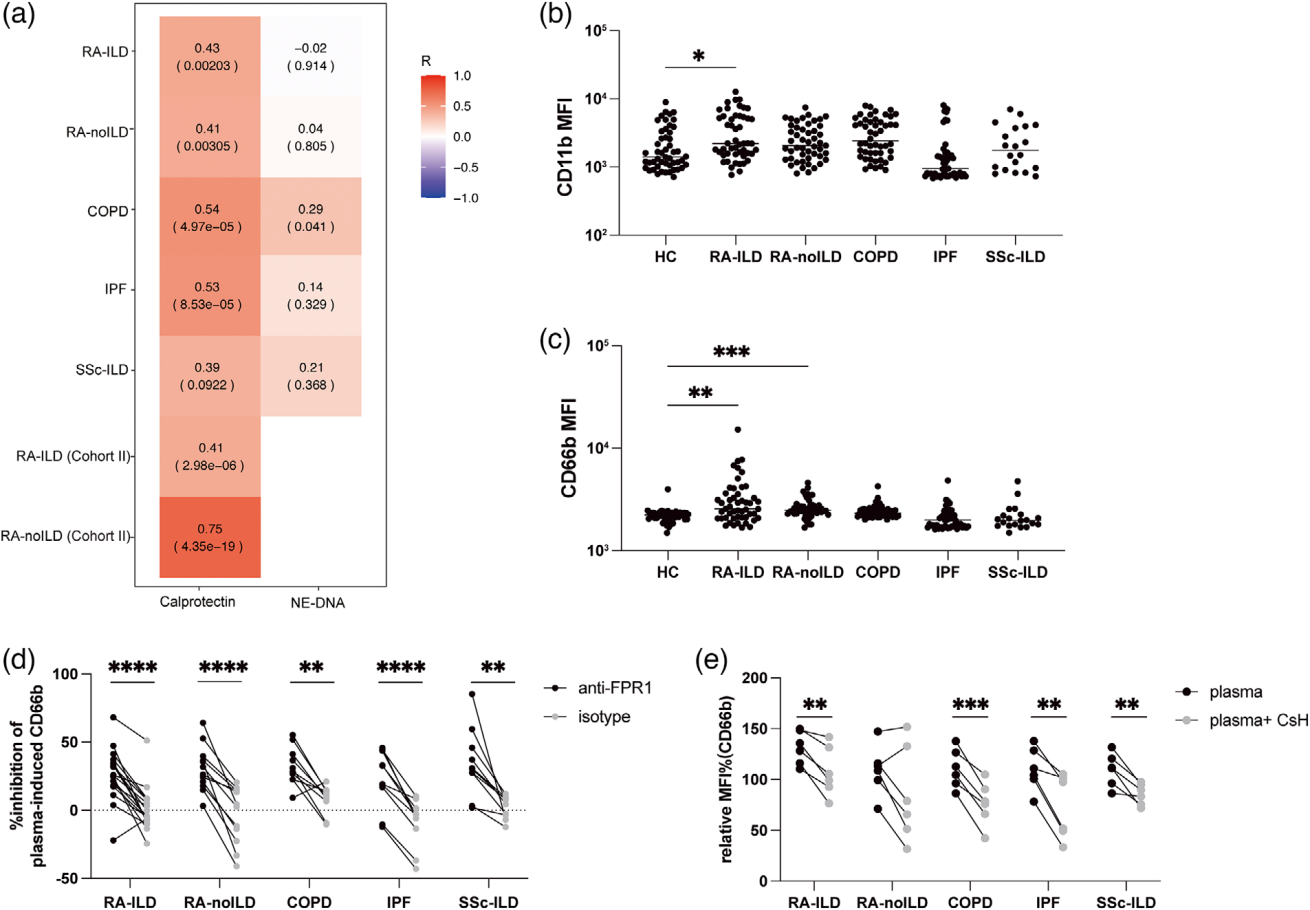
FPR1‐mediated neutrophil activation in rheumatoid arthritis–interstitial lung disease (RA‐ILD): (a) correlation heatmap of N‐formyl methionine (fMET) and neutrophil activation markers in all disease subgroups. In each cell, the upper values represent correlation coefficients, and the lower values represent p; (b and c) plasma from healthy controls and patients were incubated with healthy neutrophils and assessed for the capacity to induce upregulation of neutrophil activation markers (b) CD11b and (c) CD66b; (d) percent inhibition of plasma‐induced neutrophil activation by anti‐formyl peptide receptor‐1 (FPR1); (e) inhibition of plasma‐induced neutrophil CD66b expression by cyclosporine H (CsH). Statistical analyses by the Kruskal–Wallis test, the Mann–Whitney U test, and Spearman's correlation, with *p < 0.05, **p < 0.01, ***p < 0.001, and ****p < 0.0001. COPD, chronic obstructive pulmonary disease; IPF, idiopathic pulmonary fibrosis; SSc–ILD, systemic sclerosis–related interstitial lung disease.

fMET is a significant neutrophil agonist and can induce substantial neutrophil activation through FPR1. In our study, plasma fMET levels showed a strong correlation with calprotectin levels across all disease cohorts, suggesting an association between elevated levels of fMET and neutrophil activation (Fig. 4a). By contrast, no correlation was observed between fMET and NE‐DNA, indicating that fMET‐mediated activation may predominantly reflect degranulation rather than NET release.

To test whether patient plasma could drive neutrophil activation, neutrophils isolated from healthy controls were incubated with plasma from patients and assessed for activation markers CD11b and CD66b expression. Plasma from RA‐ILD patients induced significant upregulation of both markers (CD11b: *p *= 0.049; CD66b: *p *= 0.003. Fig. 4b,c). In RA‐noILD, plasma did not increase CD11b versus healthy controls but significantly increased CD66b (*p* = 0.0008). However, neither activation marker differed between RA‐ILD and RA‐noILD. Preincubation of neutrophils with anti‐FPR1 antibody or with CsH significantly reduced plasma‐induced activation across groups (Fig. 4d,e), demonstrating that these effects were mediated, at least in part, through FPR1 signaling.

Together with the markedly elevated plasma fMET levels in RA‐ILD, these results indicate that although RA itself can promote systemic neutrophil activation, fMET–FPR1‐dependent activation is enriched in RA‐ILD.

### Hierarchical cluster analysis of neutrophil activation in RA

**Fig. 5:**
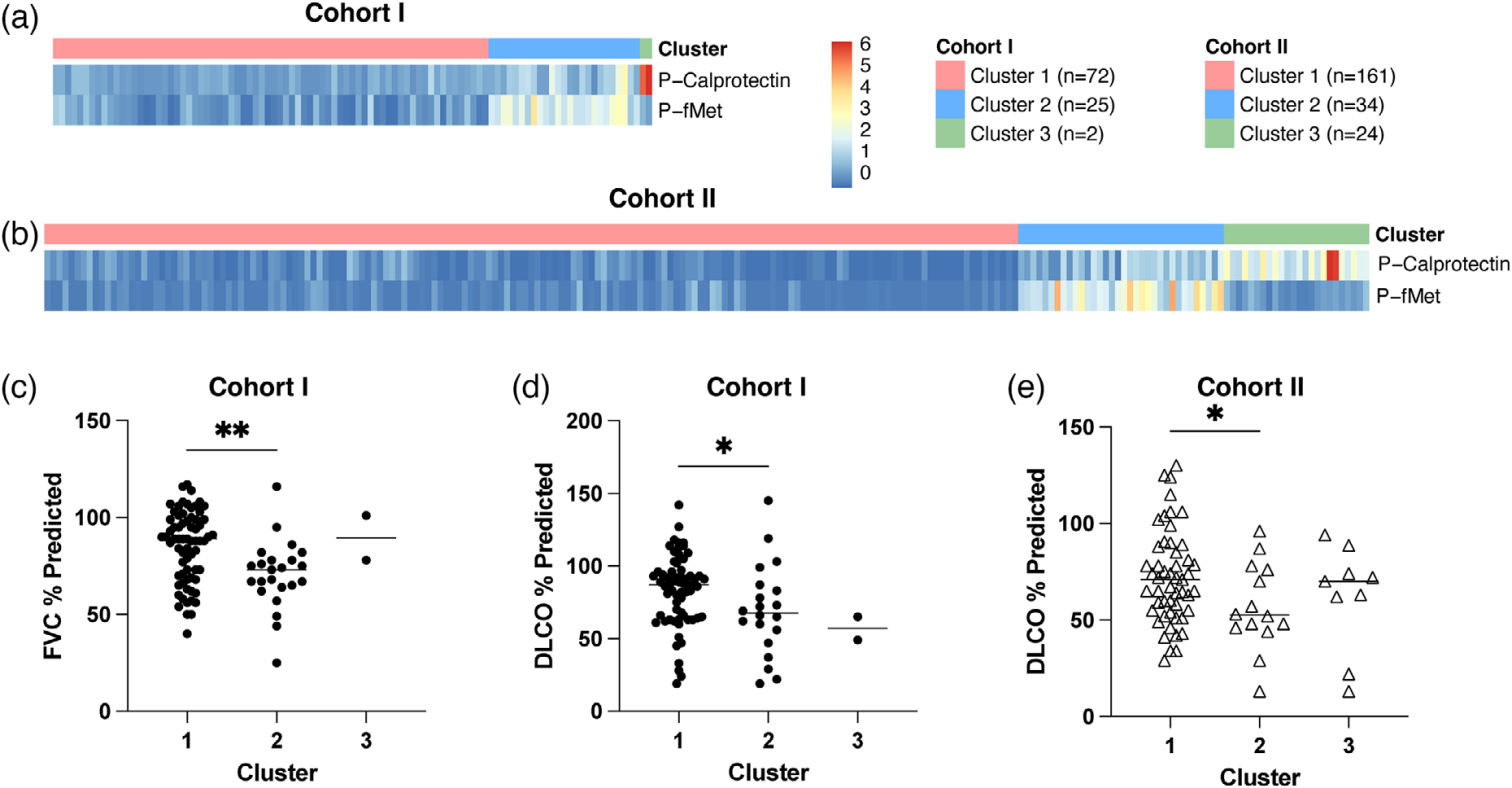
Hierarchical cluster analysis of rheumatoid arthritis (RA) patients: (a and b) heatmaps showing three clusters of RA patients identified in Cohort I (a) and Cohort II (b). (c, d) % predicted forced vital capacity (FVC) (c) and diffusing capacity for carbon monoxide (DLCO) (d) values for RA across the three clusters in Cohort I; (e) % predicted DLCO for rheumatoid arthritis–interstitial lung disease (RA‐ILD) patients in Cohort II. Note: In Cohort II, pulmonary function data are unavailable for RA‐noILD patients; therefore, only RA‐ILD patients are included in panel (e). Statistical analyses by the Mann–Whitney U test with *p < 0.05 and **p < 0.01.

To explore biomarker‐defined phenotypes, we performed clustering analysis for both cohorts, incorporating the two biomarkers shared between the cohorts, calprotectin, and fMET. Cohort I included 99 patients with RA, whereas Cohort II comprised 219 patients. The silhouette method identified three optimal clusters, which were consistently observed across both datasets (Fig. 5a,b, Table S11).

Cluster 1, the largest subgroup, was characterized by the lowest levels of both calprotectin and fMET, hence termed the “dual‐low cluster.” Cluster 2 exhibited high fMET levels with moderately elevated calprotectin levels, defining it as the “high‐fMET cluster.” Cluster 3, the smallest subgroup, displayed the highest levels of calprotectin and was labeled the “high‐calprotectin cluster.”

Pulmonary function differed significantly across the clusters (Fig. 5c,e). In Cohort I, the high‐fMET cluster exhibited lower FVC and DLCO compared to the dual‐low cluster (FVC: *p* = 0.0015; DLCO: *p* = 0.0378). Similarly, in Cohort II, the high‐fMET cluster had higher DLCO than the dual‐low cluster (*p* = 0.0474). These findings suggest a potential role of fMET in ILD development. No significant differences in pulmonary function progression were observed, likely due to limited follow‐up data.

## Discussion

Our data show that neutrophil activation is markedly elevated in both the blood and sputum of patients with lung involvement, particularly in those with RA‐ILD. Plasma from RA‐ILD patients induced robust neutrophil activation in vitro, which was attenuated by inhibition of FPR1, implicating the fMET–FPR1 axis in this process. These findings support a potential role for fMET‐mediated neutrophil activation in RA‐ILD and highlight FPR1 signaling as a possible target for future studies.

In humans, mitochondria are the sole source of endogenous fMET, which initiates mitochondrial protein synthesis and generates formyl peptides that serve as ligands for FPRs [19]. Among these, FPR1 and FPR2 are widely expressed but most abundant on neutrophils, where their engagement triggers calcium release, migration, and activation [20, 21]. fMET‐mediated neutrophil activation has been extensively documented, and in RA, neutrophils are primed for heightened responses, including increased oxidative burst, partly due to TNF‐α‐mediated upregulation of FPR1 [22, 23]. Elevated circulating fMET levels in RA correlate with neutrophil‐mediated inflammation, systemic tissue damage, and disease progression, including rheumatoid nodules [13]. Similarly, increased fMET and enhanced neutrophil activation have been linked to distinct phenotypes in SSc [24]. Collectively, these findings highlight FPR1‐mediated neutrophil activation as a critical factor in rheumatic diseases, though its role in ILD comorbidity remains unclear.

In our study, neutrophil activation in RA‐ILD, reflected by elevated levels of calprotectin in both plasma and sputum, was significantly higher than in RA‐noILD. Circulating fMET displayed a similar trend, underscoring a role in lung involvement in RA. Similar elevations of fMET were also observed in COPD, IPF, and SSc‐ILD groups, suggesting shared pathological mechanisms. However, plasma from IPF and SSc‐ILD did not significantly activate neutrophils, possibly reflecting lower bioactivity due to chemical modifications or degradation. Consistent with this notion, prior work has demonstrated that peptide composition can affect FPR1 signaling [25, 26], with ND3, ND4, ND5, ND6, and Cox I peptides generating a high FPR1 signal, whereas others (e.g., ND2) do not [26].

There are several plausible explanations for the lack of correlation between neutrophil activation markers (calprotectin, fMET) and pulmonary function within the RA‐ILD group. First, RA‐ILD is heterogeneous, encompassing fibrotic (usual interstitial pneumonia) and inflammatory (nonspecific interstitial pneumonia) subtypes, which may differ in the extent to which neutrophil‐driven inflammation contributes to functional impairment. Second, RA‐ILD pathogenesis likely involves multifactorial mechanisms, including immune complex deposition [27] and T‐cell‐mediated responses [28], which may dilute marker–function relationships. In contrast, stronger correlations observed in IPF and SSc‐ILD groups may reflect a greater reliance on neutrophil‐driven inflammation in these diseases. Interestingly, significant inverse correlations were observed in the total RA cohort, with a trend also seen in RA‐noILD patients (FVC vs. fMET, *r* = −0.30, *p *= 0.055, Fig. S1b). This raises the possibility that systemic neutrophil activation exerts subtle effects on pulmonary function even in patients without HRCT‐detected ILD, potentially relevant to early or subclinical stages of RA‐ILD pathogenesis.

Notably, elevated levels of fMET or calprotectin in RA‐noILD patients might indicate an increased risk of future ILD development. Although our study lacked longitudinal data to validate this hypothesis, these markers hold potential as early predictors for ILD progression. Supporting this, our prior work demonstrated that calprotectin levels could predict disease progression, including the development of erosive disease and extra‐articular nodules [6]. Future research should validate these findings to facilitate earlier detection and timely intervention, ultimately improving patient outcomes.

Further, to investigate the predictive value of neutrophil activation markers and fMET, we analyzed their correlations with pulmonary function progression. Interestingly, baseline plasma NE‐DNA levels showed a significant positive correlation with DLCO progression in the RA‐ILD, total RA, and IPF groups (Fig. 2). In contrast, no significant correlations were found between sputum NE‐DNA and DLCO progression or baseline DLCO (Table S4). Previous studies have predominantly highlighted the detrimental effects of NE in lung diseases, where NE promotes pro‐inflammatory responses, damages pulmonary epithelium and endothelium, and disrupts tissue homeostasis in pneumonia, asthma, and PF [29, 30, 31, 32]. NE inhibition attenuates PF in murine models by suppressing TGF‐β1 signaling and inflammatory cell recruitment [33], consistent with the traditional view of neutrophil activation as a driver of lung injury and fibrosis. However, our findings suggest a more nuanced role of NE‐DNA. NETs, with NE‐DNA as a key component, have been shown to promote tissue remodeling and repair in certain contexts, such as retinal vascular repair [34], raising the possibility that plasma NE‐DNA may similarly contribute to reparative processes in the lung. An alternative, nonmutually exclusive explanation is that higher NE‐DNA levels may reflect better treatment responsiveness. NE‐DNA (and calprotectin) has been reported as biomarkers of response to immunosuppressive therapy in RA [35]. Moreover, as NET levels have also been linked to higher RA disease activity in previous studies [6], patients with elevated NE‐DNA might have received more intensive therapy, which could in turn contribute to the observed improvement in DLCO.

Interestingly, sputum fMET levels in RA‐ILD patients were not elevated and were even slightly lower than in healthy controls (Fig. S1c). Combined with the absence of correlations between sputum and plasma markers (Table S3), this supports the notion that airway neutrophil activation is compartmentalized rather than directly reflected in systemic activity. Airway analysis, therefore, remains important in RA pathogenesis, consistent with evidence that mucosal immune activation, including NET and ACPA formation, contributes to disease initiation [36, 37]. Future studies incorporating bronchoalveolar lavage fluid or lung tissue samples will be needed to better delineate the contribution of local versus systemic neutrophil activation.

Our hierarchical clustering analysis identified three distinct reproducible subgroups based on fMET and calprotectin, underscoring the potential clinical utility of biomarker‐based stratification in RA. The largest “dual‐low cluster” reflected a low‐inflammatory phenotype, whereas the “high‐fMET” and “high‐calprotectin” clusters indicated distinct pathways of neutrophil activation: the former linked to neutrophil recruitment and activation via elevated fMET and the latter associated with tissue inflammation and damage via increased calprotectin. Importantly, both high‐marker clusters were enriched for RA‐ILD in Cohort I, linking fMET‐ and calprotectin‐driven mechanisms to lung involvement. The high‐fMET cluster had significantly lower DLCO compared to the dual‐low cluster, consistent with fMET's role in neutrophil recruitment and lung inflammation [17]. Conversely, the dual‐low cluster may indicate a more favorable phenotype with preserved pulmonary function. The small sample size of the high‐calprotectin cluster limited the statistical comparisons. Validation in larger, longitudinal cohorts will be needed to establish the predictive value of these biomarker clusters for disease progression and treatment response.

Our study faced several limitations. First, the cross‐sectional design and limited follow‐up data restricted our ability to assess the predictive role of fMET and neutrophil activation markers for RA‐ILD progression. Second, incomplete clinical data, particularly limited disease activity measures in Cohort I, lack of detailed treatment information, and small seronegative subgroups, reduced the power and generalizability of stratified analyses. Third, smoking, an established trigger of neutrophil activation [38], was recorded only qualitatively, precluding robust analysis. Future longitudinal studies with more complete clinical and exposure data are needed to confirm our findings.

## Conclusions

In summary, our study suggests a potential contribution of the fMET–FPR1 signaling pathway in neutrophil activation in RA‐ILD and highlights the immunological heterogeneity underlying RA. By defining inflammatory phenotypes based on plasma fMET and calprotectin levels, we identified systemic biomarker patterns correlated with pulmonary impairment. These findings may help refine risk stratification and surveillance of lung involvement in RA, and they provide a rationale for future studies to validate the clinical relevance and therapeutic potential of targeting the fMET–FPR1 axis.

## Author contributions

**Christian Lood**: Conceptualization; initial manuscript writing; supervision; funding acquisition. **Dan Ke**: Patient information acquisition; manuscript review and editing. **Xiaomin Liu**: Patient information acquisition; manuscript review and editing. **Xinping Tian**: Patient information acquisition; manuscript review and editing. **Mengtao Li**: Patient information acquisition; manuscript review and editing. **M. Kristen Demoruelle**: Patient information acquisition; manuscript review and editing. **Joshua J. Solomon**: Patient information acquisition. **Jia Shi**: Experiment; data analysis and interpretation; initial manuscript writing. **Xueting Yuan**: Experiment; manuscript review and editing. **Yiyun Pang**: Experiment; manuscript review and editing. **Yang Wu**: Experiment; data analysis and interpretation; manuscript review and editing. **Ting Wang**: Experiment; data analysis and interpretation; manuscript review and editing. **Ryan Stultz**: Experiment; manuscript review and editing. **Chen Yu**: Data analysis and interpretation; initial manuscript writing. **Qian Wang**: Initial manuscript writing; supervision; funding acquisition.

## Conflict of interest statement

The authors declare no conflicts of interest.

## Funding information

This study was supported by the Chinese National Key Technology R&D Program, Ministry of Science and Technology (2022YFC2504600/3/4), CAMS Innovation Fund for Medical Sciences (CIFMS) (2023‐I2M‐C&T‐B‐035, 2023‐I2M‐2‐005), and National High‐Level Hospital Clinical Research Funding (2022‐PUMCH‐C‐020), an investigator‐initiated award from Boehringer Ingelheim Pharmaceuticals, Inc. to C.L., as well as Arthritis National Research Foundation award (#632002) to C.L. C.L. is a Herndon and Esther Maury Endowed Professor of Rheumatoid Arthritis, with the endowment supporting some of the salary for this study.

## Ethics statement

The study was approved by the ethics boards of University of Washington (#3100) and PUMCH (#I‐23PJ895), and informed consent was obtained from all participants in accordance with the Helsinki Declaration.

## Disclosure

This was an independent investigator‐initiated study supported by Boehringer Ingelheim Pharmaceuticals, Inc. (BIPI). BIPI had no role in the design, analysis, or interpretation of the results in this study; BIPI was given the opportunity to review the manuscript for medical and scientific accuracy as it relates to BIPI substances, as well as intellectual property considerations.

## Patient and public involvement

Patients or the public were not involved in the design, conduct, reporting, or dissemination plans of our research.

## Supporting information




**Figure S1: Analysis of fMET in Cohort I**. (a) Comparison of % predicted FVC between RA patients with high versus low plasma fMET (threshold: mean + 2SD of healthy controls); (b) correlation between plasma fMET levels and % predicted FVC in RA‐noILD; (c) comparison of sputum fMET levels among healthy controls, RA‐ILD, and RA‐noILD patients. Statistical analyses by the Mann–Whitney *U* test and Spearman's correlation with *p < 0.05 and **p < 0.01.

**Table S1**: Overview of biomarker, serology, and clinical data availability in Cohorts I and II.
**Table S2**: Comparisons of plasma calprotectin levels between RA‐ILD and RA‐noILD after adjustment for serological status (Cohort I) or disease activity (Cohort II).
**Table S3**: Correlations between plasma and sputum neutrophil activation markers in Cohort I.
**Table S4**: Correlation analysis between pulmonary function parameters, neutrophil activation markers, and fMET.
**Table S5**: Correlation and regression analyses of plasma calprotectin with pulmonary function in Cohort I after adjustment for seropositivity.
**Table S6**: Correlation and regression analyses of plasma calprotectin with pulmonary function in Cohort II after adjustment for disease activity.
**Table S7**: Correlation and regression analyses of plasma NE‐DNA with pulmonary function progression in Cohort I after adjustment for seropositivity.
**Table S8**: Comparisons of plasma fMET levels between RA‐ILD and RA‐noILD after adjustment for serological status (Cohort I) or disease activity (Cohort II).
**Table S9**: Correlation and regression analyses of plasma fMET with pulmonary function in Cohort I after adjustment for seropositivity.
**Table S10**: Correlation and regression analyses of plasma fMET with pulmonary function in Cohort II after adjustment for disease activity.
**Table S11**: Comparison of clinical characteristics of the three clusters in two independent cohorts.

## Data Availability

The data supporting the findings of this study are available from the corresponding author upon reasonable request.
