# Supplementary material for: Systemic neutrophil activation and N‐formyl methionine‐formyl peptide receptor‐1 signaling define inflammatory endotypes in rheumatoid arthritis‐associated lung involvement
Source: J Intern Med. 2025 Oct 14;298(6):656–69. doi: 10.1111/joim.70030 (PMC12617476; doi:10.1111/joim.70030)
Supplement: Supplementary file 1 — Figure S1: Analysis of fMET in Cohort I. (a) Comparison of % predicted FVC between RA patients with high versus low plasma fMET (threshold: mean + 2SD of healthy controls); (b) correlation between plasma fMET levels and % predicted FVC in RA‐noILD; (c) comparison of sputum fMET levels among healthy controls, RA‐ILD, and RA‐noILD patients. Statistical analyses by the Mann–Whitney U test and Spearman's correlation with *p < 0.05 and **p < 0.01. [file JOIM-298-656-s002.docx]

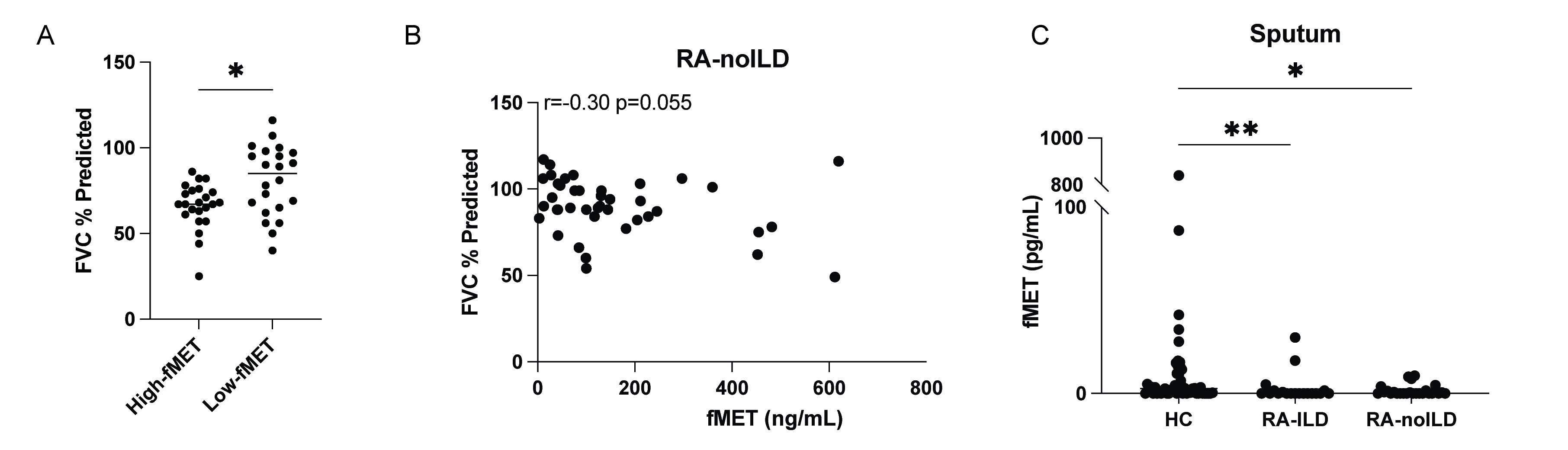


**Figure S1 Analysis of fMET in Cohort I.** (A) Comparison of % predicted FVC between RA patients with high versus low plasma fMET (threshold: mean + 2SD of healthy controls). (B) Correlation between plasma fMET levels and % predicted FVC in RA-noILD. (C) Comparison of sputum fMET levels among healthy controls, RA-ILD, and RA-noILD patients. Statistical analyses by Mann-Whitney U test and Spearman’s correlation with * p<0.05 and ** p<0.01.
